# Supplementary material for: Therapeutic 6-thio-deoxyguanosine inhibits telomere elongation in cancer cells by inducing a non-productive stalled telomerase complex
Source: Nat Commun. 2025 Dec 7;17:6. doi: 10.1038/s41467-025-66534-w (PMC12764492; doi:10.1038/s41467-025-66534-w)
Supplement: Supplementary file 1 — Supplementary Information [file 41467_2025_66534_MOESM1_ESM.pdf]

Supplementary Information

Supplementary Table 1: Oligonucleotides sequences 5' to 3'

Name	Sequence
**Primer 1**	GGTTAGGGTTAGGGTTAG
**Primer 2**	GGTTAGGGTTAGGGTTA/6dG/
**Primer 3**	GGTTAGGGTTAGG/6dG/TTAG
**Primer 4**	GTTAGGGTTAGGGTTAGG
**Primer 5**	GTTAGGGTTAGGGTTAG/6dG/
**Primer 6**	GTTAGGGTTAGGGTTA/6dG/G
**Primer 7**	GTTAGGGTTAGGGTTAG/6dG/6dG/
**Primer A5**	TTAGGGTTAGCGTTAGGG
**EMSAoligo1**	/5Phos/TTCAGAG/iFluorT/CTGACGGTTAGGGTTAGGGTTAG
**EMSAoligo2**	/5Phos/TTCAGAG/iFluorT/CTGACGGTTAGGGTTAGGGTTA/6dG/
**EMSAscramble**	/5Phos/TTCAGAG/iFluorT/ CTGACGGCTGCTACCTACGGCCTT
**hTR dot blot probe**	CGGTGGAAGGCGGCAGGCCGAGGC
**smFRET primer**	Biotin/TTAGGGTTAGGG/5AmMC6 T/TAGGG

6dG depicts 6-thio-dG; iFluor depicts fluorescein; 5AmMC6 depicts 5' amino modifier C6

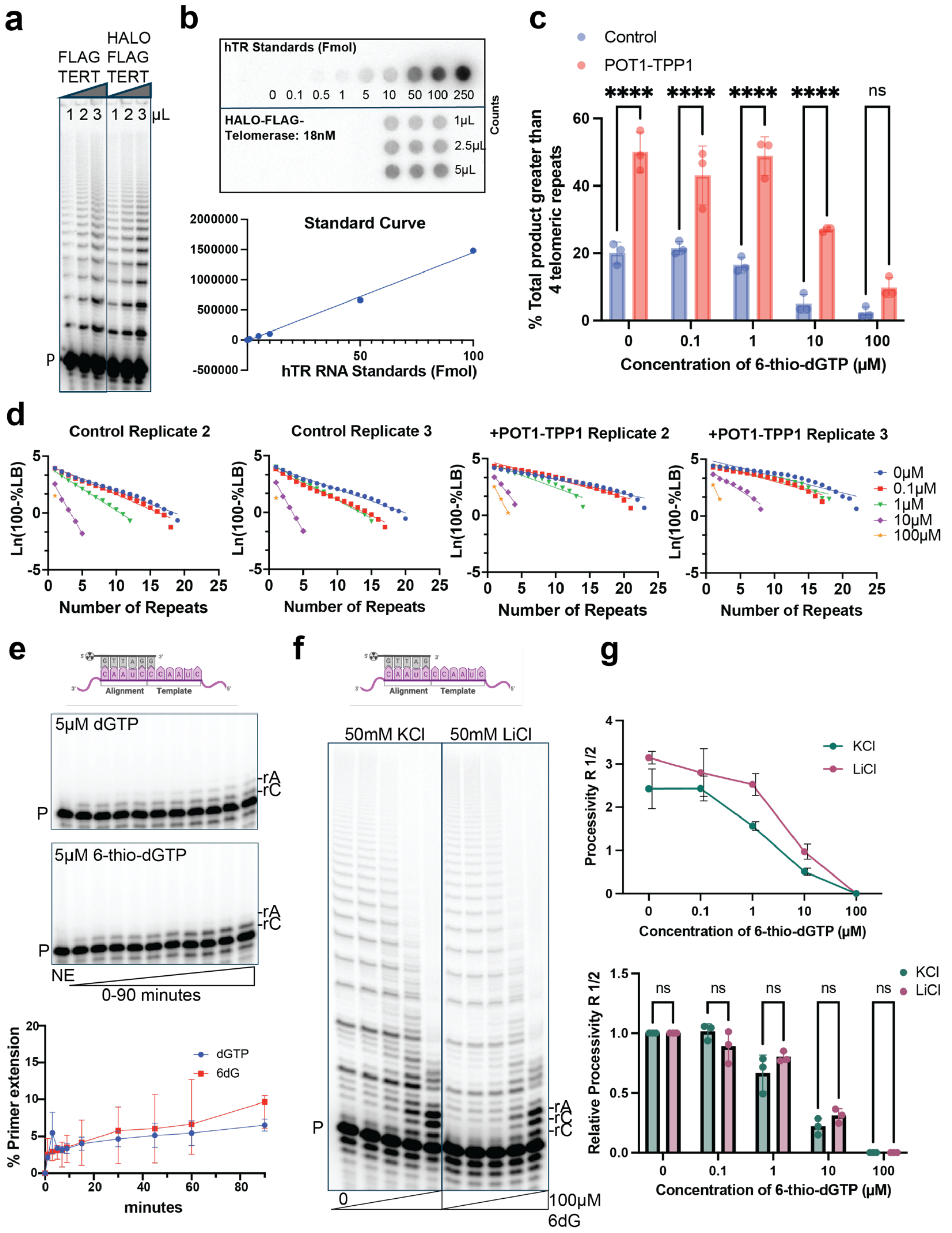
a
FLAG TERT
HALO FLAG TERT
1 2 3 1 2 3 μL
P
b
hTR Standards (Fmol)
0 0.1 0.5 1 5 10 50 100 250
HALO-FLAG-Telomerase: 18nM
1μL
2.5μL
5μL
Counts
Standard Curve
hTR RNA Standards (Fmol)
c
Control
POT1-TPP1
% Total product greater than 4 telomeric repeats
Concentration of 6-thio-dGTP (μM)
**** **** **** **** ns
d
Control Replicate 2
Control Replicate 3
+POT1-TPP1 Replicate 2
+POT1-TPP1 Replicate 3
Ln(100-%LB)
Number of Repeats
0μM
0.1μM
1μM
10μM
100μM
e
Alignment
Template
5μM dGTP
5μM 6-thio-dGTP
rA
rC
P
NE
0-90 minutes
% Primer extension
dGTP
6dG
minutes
f
50mM KCl
50mM LiCl
rA
rC
rC
P
0
100μM 6dG
g
Processivity R 1/2
KCl
LiCl
Concentration of 6-thio-dGTP (μM)
Relative Processivity R 1/2
ns ns ns ns ns

Supplementary Fig. 1. Quantification of immunopurified telomerase (hTERT/hTR) complexes. a, Direct telomerase assay conducted with FLAG-tagged telomerase (lanes 1-3) and Halo-FLAG-tagged telomerase (HALO, lanes 4-6) and 5 nM ^{32}P-end labeled primer (GGTTAGGGTTAGGGTTAG) (Primer 1, Supplementary Table 1). Reactions contained cellular-concentration dNTPs. P indicates unextended primer. **b,** Telomerase was over expressed in HEK293T cells and immunopurified using a FLAG tag on the N-terminus of TERT. The Halo-FLAG telomerase was spotted on a blot and probed with a ^{32}P labeled probe against hTR (see Supplementary Table 1) to determine the concentrations of RNA that co-purified with Halo-FLAG-tagged telomerase. 1, 2.5, and 5 μL aliquots were compared with in vitro transcribed hTR standards from 0.5 to 250 fmol. Concentrations are shown. Bottom panel shows standard curve of hTR standards to determine concentration of telomerase. **c**, quantification of % total products extended greater than 4 repeats from reactions in Fig. 1b. Data represent the mean ± s.d. from three independent experiments. ns = not significant; ****$P<0.0001$ two-way ANOVA. **d**, Processivity (R 1/2) calculated on the basis of total products normalized to loading control shown for replicates 2 and 3. **e**, Direct telomerase assays were conducted with 5 nM ^{32}P-end labeled Primer 4 (Supplementary Table 1) and 5 μM dGTP. Reaction aliquots were removed at 1, 3, 5, 7, 9, 15, 30, 45, 60 and 90 s and terminated with 0.5M EDTA. Letters on the right indicate template base and P indicates unextended 18-mer primer. NE indicted no enzyme control. Bottom panel show quantification of % primer extension. Data represent the mean ± s.d. from three independent experiments. **f**, Direct telomerase assays were conducted with 5 nM ^{32}P-end labeled Primer 1 (Supplementary Table 1) and cellular-concentration dNTP for 60 min. Reactions contained 0-100μM 6-thio-dGTP and either 50 mM KCl or 50 mM LiCl as indicated. Letters on the right indicate template base and P indicates unextended primer. Created in BioRender. Sanford, S. (2025) https://BioRender.com/0jpk3xg

g, Top panel, processivity (R 1/2) calculated on the basis of total products normalized to loading control. Data represent the mean ± s.d. from three independent experiments. Bottom panel shows relative processivity compared to 0 μM 6-thio-dGTP for reactions conducted in KCl or LiCl at each 6-thio-dGTP concentration. Data represent the mean ± s.d. from three independent experiments. ns = not significant, two-way ANOVA. Source data are provided as a Source Data file.

a

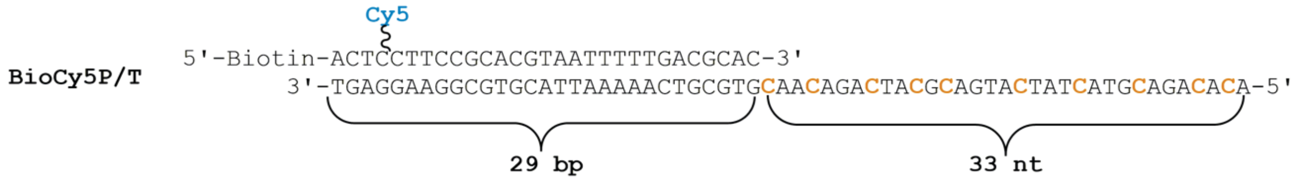

Cy5
BioCy5P/T
5'-Biotin-ACTCCTTCCGCACGTAATTTTTGACGCAC-3'
3'-TGAGGAAGGCGTGCATTAAAAACTGCGTGCAACAGACTACGCAGTACTATCATGCAGACACA-5'
29 bp
33 nt

b

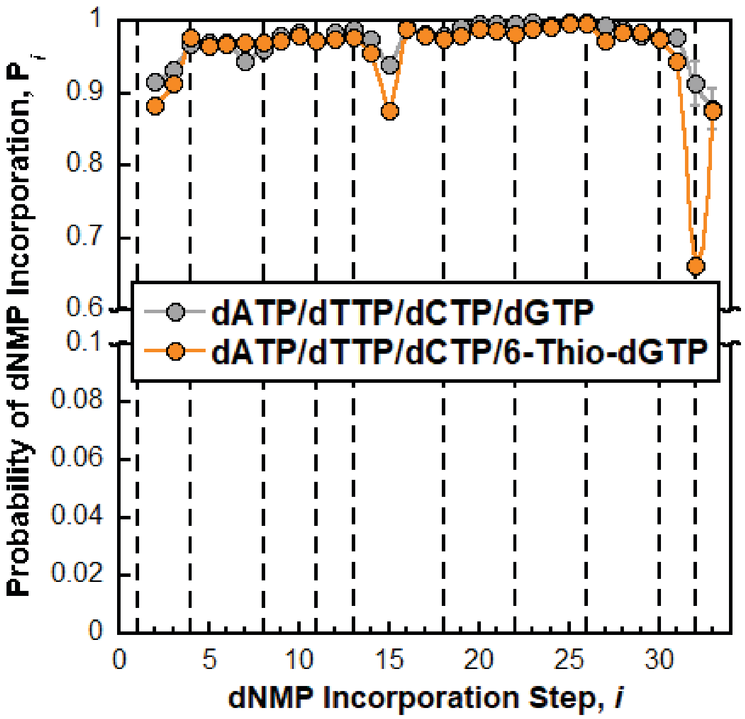

Probability of dNMP Incorporation, P_i
dNMP Incorporation Step, *i*
dATP/dTTP/dCTP/dGTP
dATP/dTTP/dCTP/6-Thio-dGTP
1
0.9
0.8
0.7
0.6
0.1
0.08
0.06
0.04
0.02
0
0
5
10
15
20
25
30

Supplementary Fig. 2. The DNA substrate utilized for Pol δ holoenzyme DNA synthesis. **a**, The length (29 bp) of the double strand DNA (dsDNA) region is designed to meet the requirements for assembly of a single PCNA ring onto DNA by RFC. The ssDNA (template) region adjacent to the 3' end of the Primer/Template (P/T) junction contains 10 cytosine (C) nucleotides (highlighted in orange) and accommodates one RPA molecule. RPA prevents loaded PCNA from sliding off the ssDNA end of the substrate. When pre-bound to neutravidin, the biotin attached to the 5'end of a primer strand prevents loaded PCNA from sliding off the dsDNA end of the substrate. **b**, Efficiency of DNA synthesis by pol δ holoenzymes in the presence of 6-thio-dGTP. The efficiencies of dNTP incorporation in the presence of 6-thio-dGTP are plotted as a function of dNTP incorporation step, *i*. Data points in orange indicate dNTP incorporation steps, *i*, in the presence of 6-thio-dGTP. Dotted lines indicate where the ssDNA template nucleotide is cytidine and, hence, 6-thio-dGTP is incorporated. Source data are provided as a Source Data file.

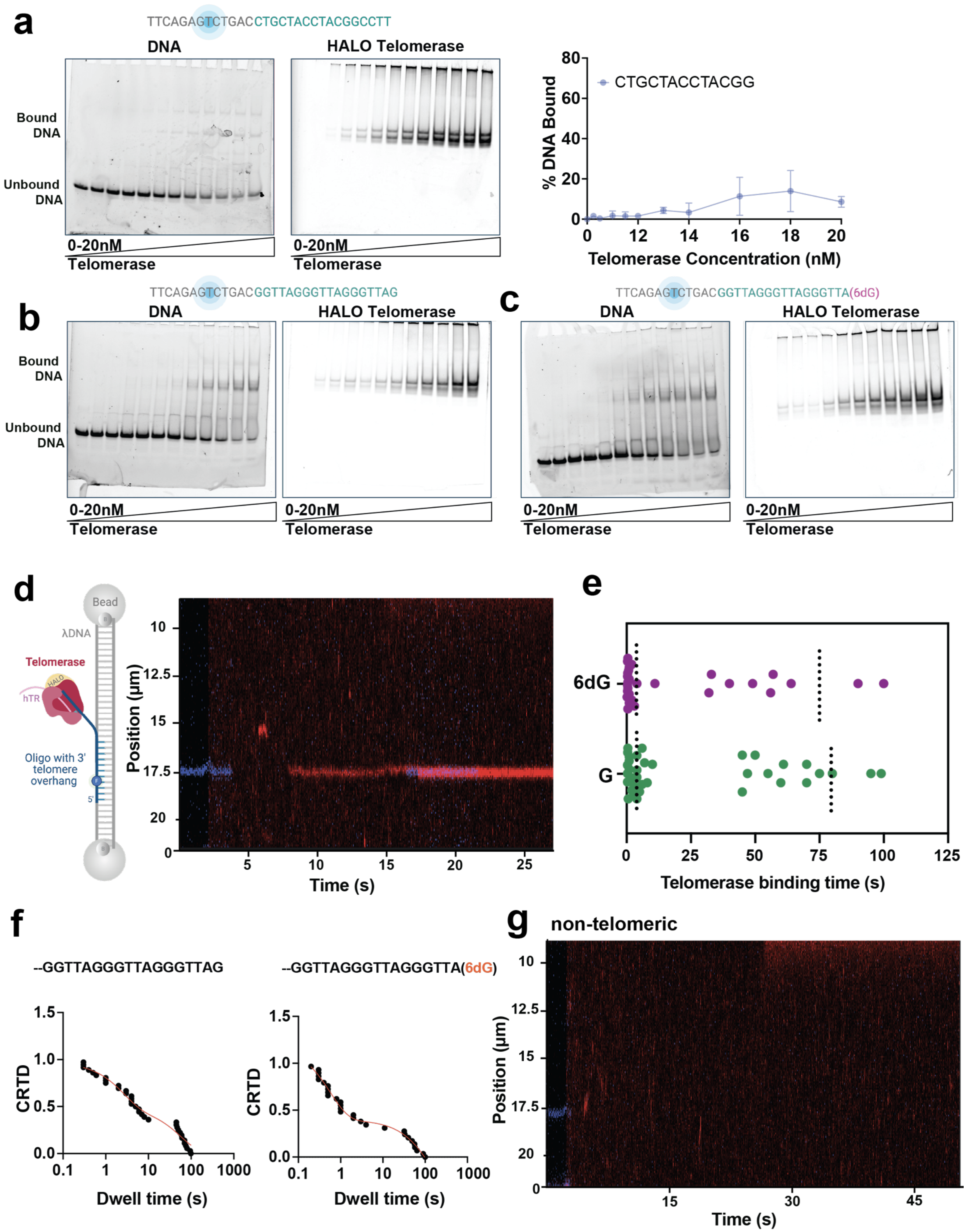

a
TTCAGAGTCTGACCTGCTACCTACGGCCTT
DNA
HALO Telomerase
Bound DNA
Unbound DNA
0-20nM Telomerase
% DNA Bound
CTGCTACCTACGG
Telomerase Concentration (nM)
b
TTCAGAGTCTGACGGTTAGGGTTAGGGTTAG
c
TTCAGAGTCTGACGGTTAGGGTTAGGGTTA(6dG)
d
Bead
λDNA
Telomerase
HALO
hTR
Oligo with 3' telomere overhang
5'
Position (μm)
Time (s)
e
6dG
G
Telomerase binding time (s)
f
--GGTTAGGGTTAGGGTTAG
--GGTTAGGGTTAGGGTTA(6dG)
CRTD
Dwell time (s)
g
non-telomeric
Position (μm)
Time (s)

Supplementary Fig. 3. Single molecule analysis of telomerase binding to telomeric substrates containing 6-thiodG. a, Binding reactions for EMSA scramble (Supplementary Table 1) were conducted with 2.5nM DNA scramble control substrate, 1-20 nM Halo-telomerase conjugated with JF-635 dye for 30 min at room temperature, and separated by EMSA to visualize telomerase-bound substrate. **b,** Quantification of % DNA bound versus Halo- telomerase concentration. Data represent the mean ± s.d. from two independent experiments. **c and d**, Black and white images for each channel: DNA and Halo-telomerase binding reactions conducted in Main Figure 3D. **e,** Cartoon of two streptavidin beads bound to lambda DNA with telomerase (red) bound to the 3' overhang (blue). Representative kymograph of telomerase (red) bound to DNA fiducial marker (blue) shown (y axis indicates position and x-axis indicates time bound). Created in BioRender. Sanford, S. (2025) https://BioRender.com/c7cn6y4. **e,** Plot of telomerase binding times bound to a telomeric overhang with and without 6-thio-dG. **f**. The CRTD plot for all dwell times (s) of primers ending in G (**d**) vs 6dG. **g**, Representative kymograph of EMSA Scramble DNA (blue) indicating no telomerase binding (red). Source data are provided as a Source Data file.

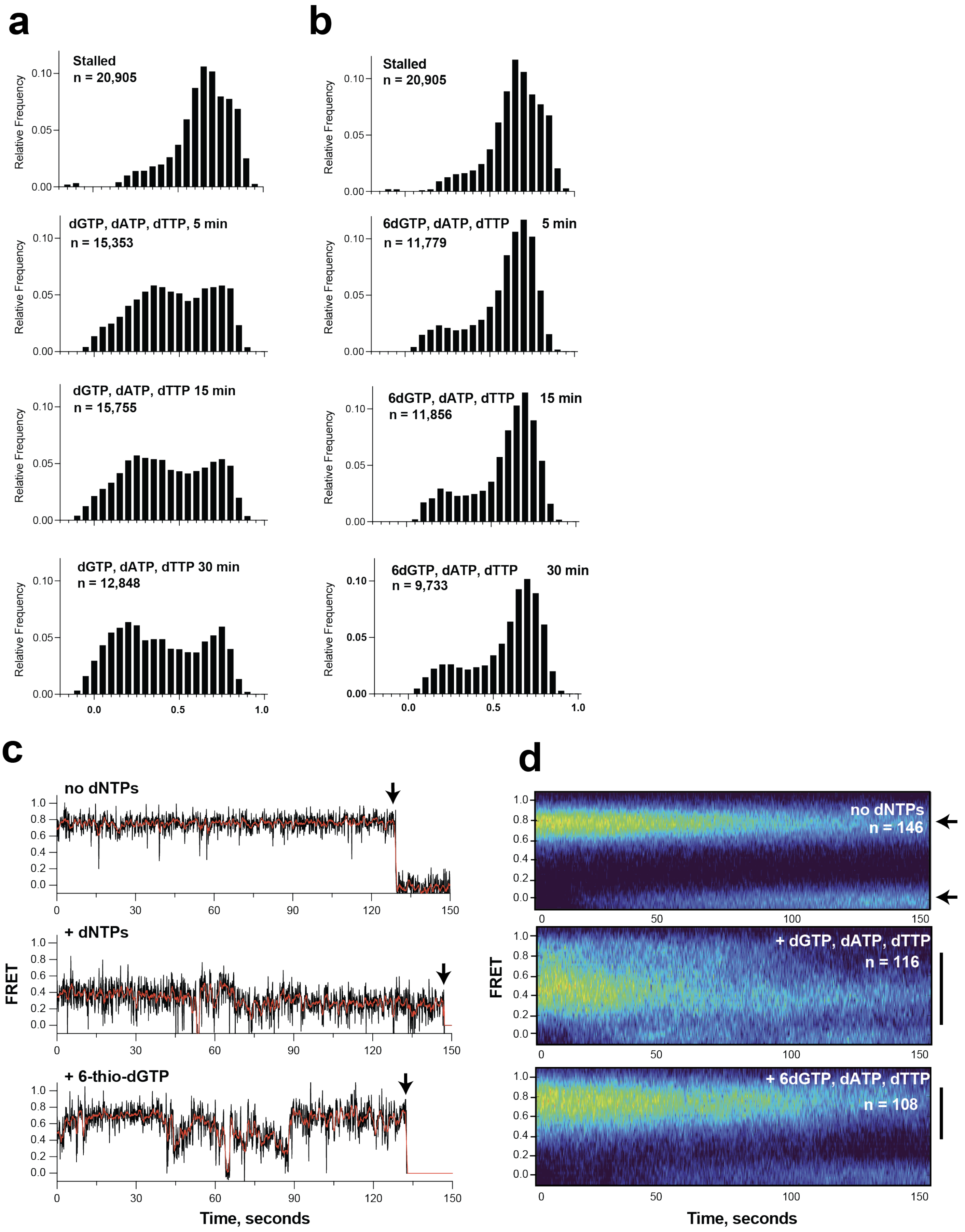

a
b
Stalled
n = 20,905
dGTP, dATP, dTTP, 5 min
n = 15,353
dGTP, dATP, dTTP 15 min
n = 15,755
dGTP, dATP, dTTP 30 min
n = 12,848
6dGTP, dATP, dTTP 5 min
n = 11,779
6dGTP, dATP, dTTP 15 min
n = 11,856
6dGTP, dATP, dTTP 30 min
n = 9,733
Relative Frequency
c
no dNTPs
+ dNTPs
+ 6-thio-dGTP
FRET
Time, seconds
d
no dNTPs
n = 146
+ dGTP, dATP, dTTP
n = 116
+ 6dGTP, dATP, dTTP
n = 108

Supplementary Fig. 4. Single molecule analysis of telomerase and DNA complexes by field view collection. Experimental data were collected from twenty unique frame movies (~2 seconds) in different fields of view for each time point (5, 15, 30 min reactions). **a**, FRET histograms of complexes prior to telomerase activity (top “stalled”), and collected 5, 15, and 30 minutes after dNTP (dATP, dTTP, and dGTP) addition as indicated. Shift from high to low FRET states reports on telomerase movement away from the acceptor dye during telomere elongation. **b**, FRET histograms of complexes collected prior to (top) and collected 5, 15, and 30 minutes after dATP, dTTP and 6-thio-dGTP (6dGTP) addition. **c**, Representative traces of stalled single telomerase-DNA complexes (top), in the presence of dNTPs (middle panel), or with dATP, dTTP and 6-thio-dGTP (bottom panel). Data were collected approximately 15 minutes after addition of dNTPs. Raw data collected at 8 Hz framerate are shown in black and a one second moving average is overlaid in red. Black arrows indicate irreversible photobleaching of the FRET dyes. **d**, Heat map analysis of the time dependent FRET signal of more than one hundred individual telomerase-DNA complexes in each experimental condition. Small black arrows in top panel indicate FRET state of stalled complex (upper arrow) and FRET when dyes have photobleached. In the presence of dNTPs (middle) or dATP, dTTP and 6-thio-dGTP (bottom), broadening of the FRET distribution (solid black lines) indicates DNA dynamics within the telomerase-DNA complexes. Source data are provided as a Source Data file.

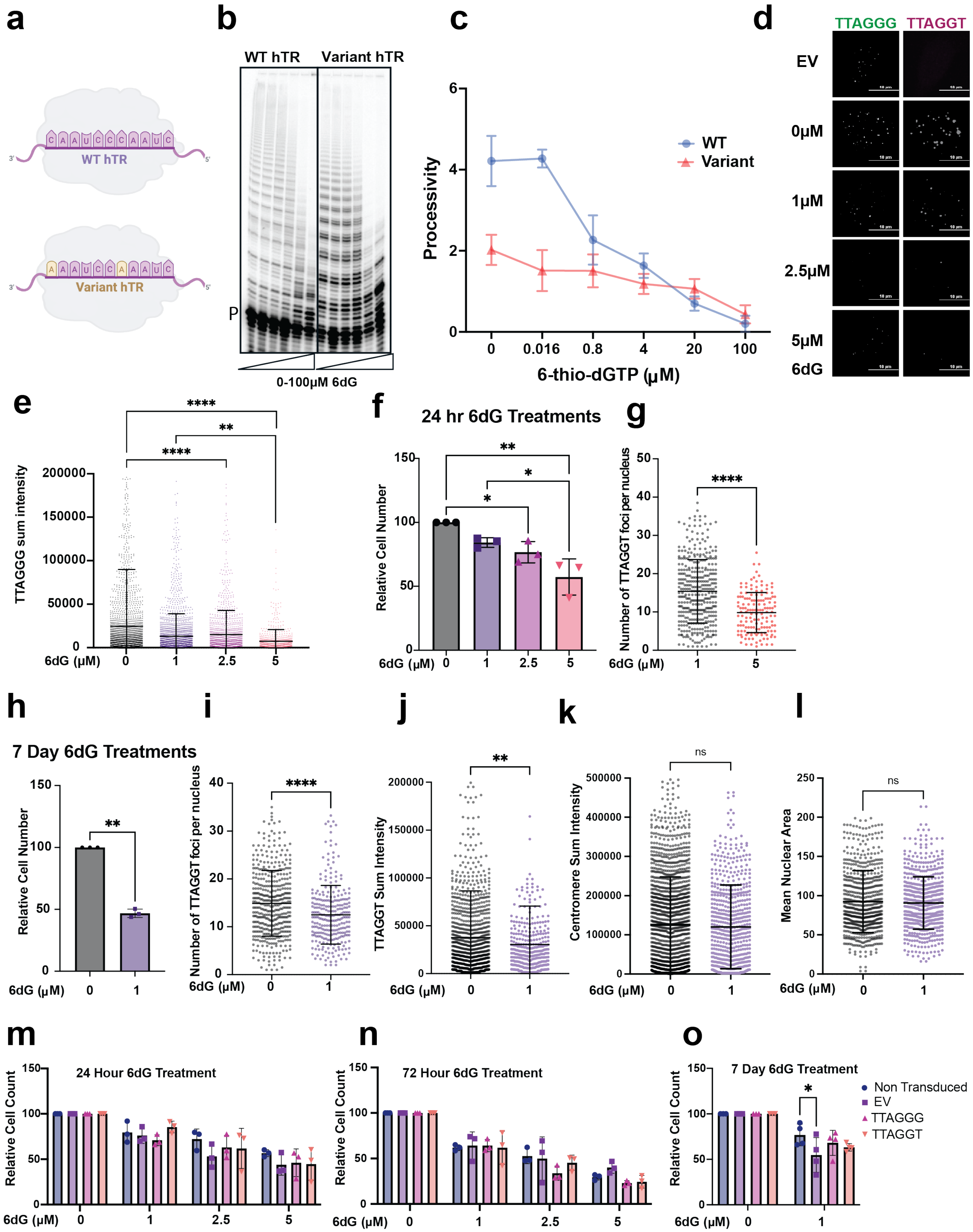
a
CAAUCCCAAUC
WT hTR
AAAUCCAAAUC
Variant hTR
3'
5'
b
WT hTR
Variant hTR
P
0-100µM 6dG
c
Processivity
WT
Variant
6-thio-dGTP (µM)
0
0.016
0.8
4
20
100
d
TTAGGG
TTAGGT
EV
0µM
1µM
2.5µM
5µM
6dG
e
TTAGGG sum intensity
****
**
****
6dG (µM)
f
24 hr 6dG Treatments
Relative Cell Number
**
*
*
6dG (µM)
g
Number of TTAGGT foci per nucleus
****
6dG (µM)
h
7 Day 6dG Treatments
Relative Cell Number
**
6dG (µM)
i
Number of TTAGGT foci per nucleus
****
6dG (µM)
j
TTAGGT Sum Intensity
**
6dG (µM)
k
Centromere Sum Intensity
ns
6dG (µM)
l
Mean Nuclear Area
ns
6dG (µM)
m
24 Hour 6dG Treatment
Relative Cell Count
6dG (µM)
n
72 Hour 6dG Treatment
Relative Cell Count
6dG (µM)
o
7 Day 6dG Treatment
Relative Cell Count
*
6dG (µM)
Non Transduced
EV
TTAGGG
TTAGGT

Supplementary Fig. 5. Inhibition of telomerase activity in cells at various 6-thio-dG treatment durations. a, Cartoons of WT and variant hTR sequences. Created in BioRender. Sanford, S. (2025) https://BioRender.com/3i6v4sh **b,** Direct telomerase assay conducted with WT and C50/56A hTR and 5 nM ^{32}P-end labeled Primer 1 (Supplementary Table 1). Reactions contained cellular-concentration dNTPs and increasing concentrations (0-100μM) of 6-thio-dGTP (6dG). P indicates unextended 18-mer primer. **c,** Telomerase processivity calculation versus 6-thio-dGTP concentration. **d**, Representative grey-scale images of interphase cells stained with PNA probes for wildtype and variant sequence 6 days post-transduction with empty vector (EV) or C50/56A hTR, and after 72 hours treatment with indicated 6-thio-dG (6dG) concentration. **e,** Quantification of wild type telomere TTAGGG repeat sum intensity. Error bars represent the mean ± s.d. of *n* variant telomere foci analyzed as indicated by dots, from 3 independent experiments. Statistical significance was determined by two-way ANOVA (1 μM vs 5μM **P = 0.0074; 2.5μM vs 5μM **P = 0.0010; ****P < 0.0001). **f,** HCT116 cells 6 days post-transduction with empty vector (EV) or C50/56A hTR were treated for 24 h with 0, 1, 2.5 or 5 μM 6-thiod-G. Cell counts obtained relative to untreated cells. Error bars represent the mean ± s.d. from 3 independent experiments. Statistical significance was determined by one-way ANOVA (0μM vs 2.5μM *P = 0.0383; 1μM vs 5μM *P = 0.0184; 0μM vs 5μM **P = 0.0012). **g,** Quantification of the number of variant (TTAGGT) telomeric foci per nuclei observed 24 h after treatment with 0 or 5 μM 6-thio-dG. Error bars represent the mean ± s.d. of *n* cells analyzed as indicated by the dots, from 3 independent experiments. Statistical significance was determined by unpaired t test with Welch's correction (****P <0.0001). **h,** HCT116 cell counts obtained 7 days after treatment with 0 or 1μM 6-thiod-G 6-thio-dG, relative to untreated cells. Error bars represent the mean ± s.d. from 3 independent experiments. Statistical significance was determined by unpaired t test with Welch's correction (**P = 0.0014). **i-l**, HCT116 cells 6 days post-transduction with empty vector (EV) or C50/56A hTR were treated for 7 days with 0 or 1 μM 6-thio-dG, and stained with variant telomere probes. Quantification of the number of variant telomere foci per nucleus (**i**), variant telomere sum intensity (**P = 0.0067) (**j**), or centromere probe sum intensity (**k**). Error bars represent the mean ± s.d. of *n* nuclei analyzed as indicated by dots, from 4 independent experiments. Statistical analysis was by two-tailed t-test (**P = 0.0067; ****P < 0.0001; n.s. = not significant). **l,** Size of mean nuclear area (μm^2) of HCT116 cells obtained 7 days after 0 or 1 μM 6-thiodG treatment. Statistical analysis by two-tailed t-test (ns = not significant). (**m-o**). HCT116 cells 6 days post-transduction with non-transduced, empty vector (EV), WT (TTAGGG) or C50/56A hTR (TTAGGT) were treated for 24 h (**m**), 72 h (**n**), or 7 days (**o**) with 0, 1, 2.5 or 5 μM 6-thio-dG. Relative cell counts were quantified from 3 independent experiments. Statistical significance was determined by one-way ANOVA (*P = 0.0145). Source data are provided as a Source Data file.

a

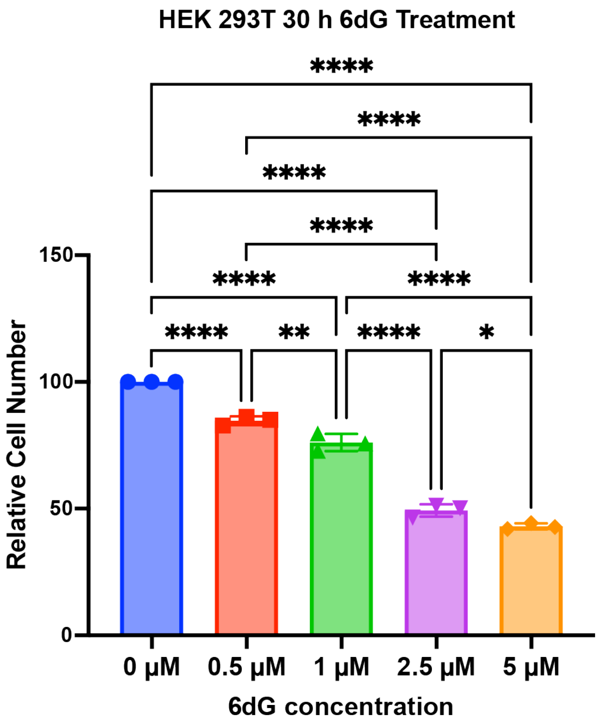
HEK 293T 30 h 6dG Treatment
****
****
****
****
****
****
****
**
****
*
150
100
50
0
Relative Cell Number
0 μM
0.5 μM
1 μM
2.5 μM
5 μM
6dG concentration

b

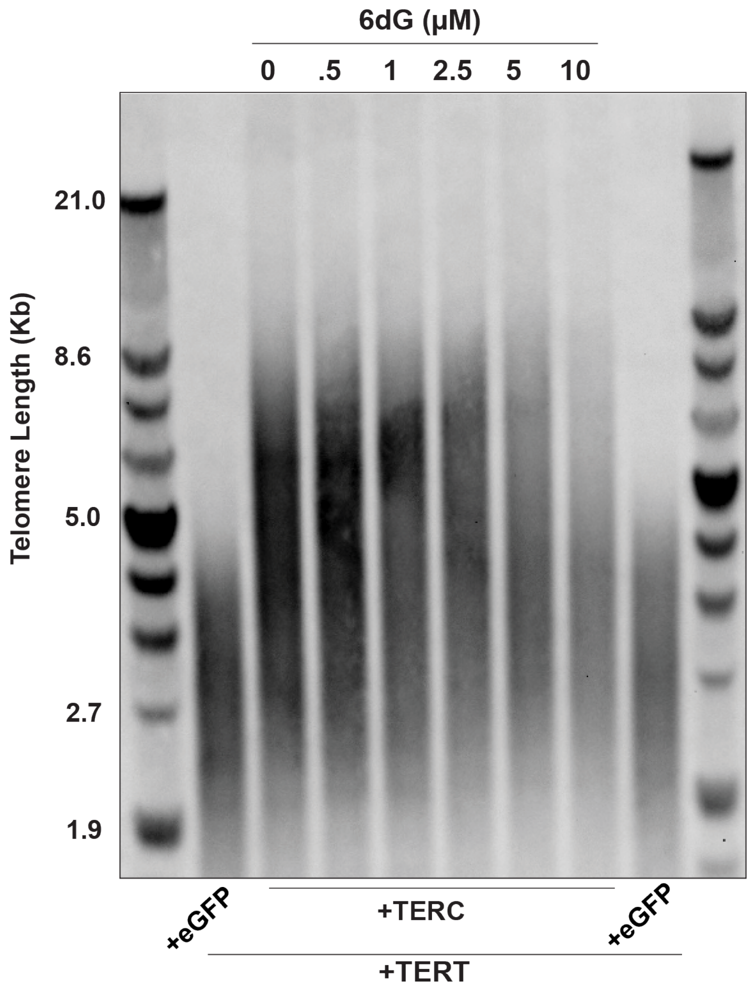
6dG (μM)
0
.5
1
2.5
5
10
21.0
8.6
5.0
2.7
1.9
Telomere Length (Kb)
+eGFP
+TERC
+eGFP
+TERT

c

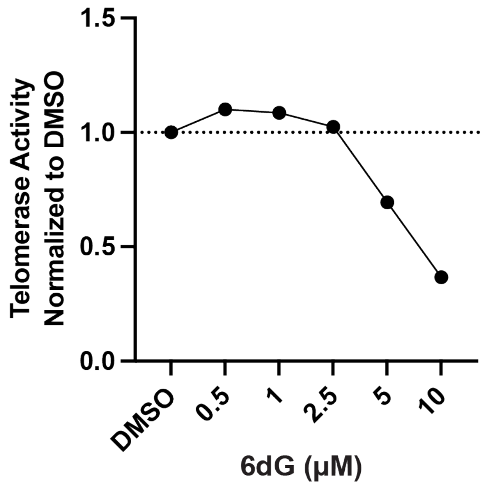
1.5
1.0
0.5
0.0
Telomerase Activity
Normalized to DMSO
DMSO
0.5
1
2.5
5
10
6dG (μM)

Supplementary Fig. 6. 6-thio-dG inhibition of telomere extension assay in cells.

a, Cell counts of HCT116 cells obtained 30 h after treatment with the indicated 6-thio-dG concentrations, relative to untreated cells. Error bars represent the mean ± s.d. from 4 independent experiments. Statistical significance was determined by one-way ANOVA (2.5μM vs 5μM *P = 0.0282; 0.5μM vs 1μM **P = 0.0038; ****P < 0.0001). **b,** Telomere restriction fragment (TRF) Southern blot of 293T hTR$^{-/-}$ cells transfected with the indicated expression vectors, cultured for 18 h and then treated with the indicated dose of 6-thio-dG for 30 h. **c**, Quantification of mean relative telomere length is shown normalized to untreated super-telomerase expressing cells. Source data are provided as a Source Data file.

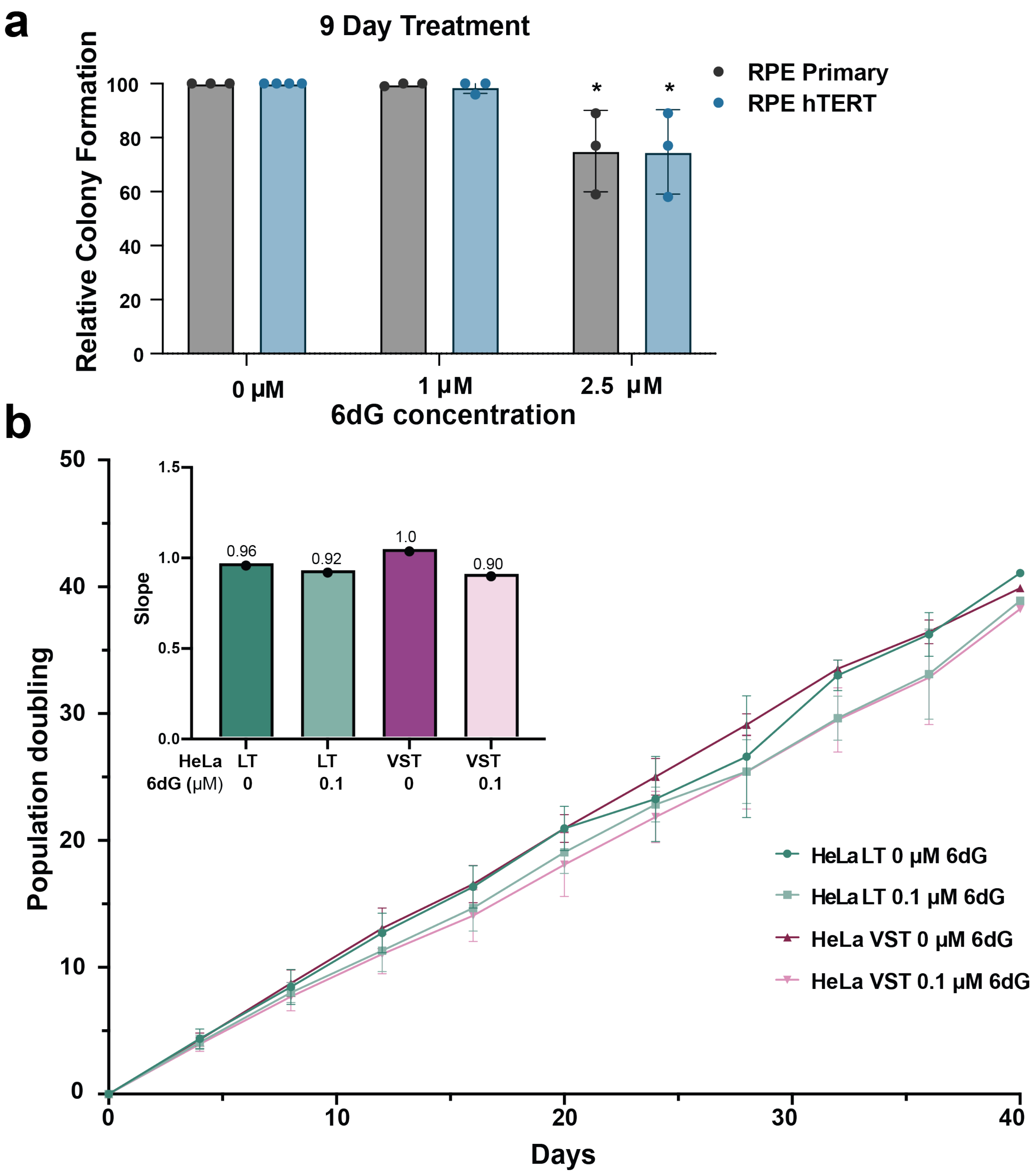
a
9 Day Treatment
Relative Colony Formation
100
80
60
40
20
0
*
*
RPE Primary
RPE hTERT
0 μM
1 μM
2.5 μM
6dG concentration
b
Population doubling
50
40
30
20
10
0
Days
0
10
20
30
40
Slope
1.5
1.0
0.5
0.0
0.96
0.92
1.0
0.90
HeLa LT LT VST VST
6dG (μM) 0 0.1 0 0.1
HeLa LT 0 μM 6dG
HeLa LT 0.1 μM 6dG
HeLa VST 0 μM 6dG
HeLa VST 0.1 μM 6dG

Supplementary Fig. 7. **Sensitivity of various cell lines to 6-thio-dG. a,** Colony formation efficiency of RPE and RPE-hTERT cells following 9 days of treatment with 1 or 2.5 μM 6-thiodG, relative to untreated cells. Error bars represent the mean ± s.d. from the number of independent experiments indicated by the dots. Statistical significance was determined by one-way ANOVA (*P = 0.0112). **b,** Population doubling of HeLa LT and HeLa VST at various time points over 20 days of no treatment or treatment with 0.1 μM 6-thiodG. Error bars represent mean ± s.d. of 3 independent experiments. Slopes of growth curves are shown in the inset. Source data are provided as a Source Data file.
