# Supplementary material for: Therapeutic 6-thio-deoxyguanosine inhibits telomere elongation in cancer cells by inducing a non-productive stalled telomerase complex
Source: Nat Commun. 2025 Dec 7;17:6. doi: 10.1038/s41467-025-66534-w (PMC12764492; doi:10.1038/s41467-025-66534-w)
Supplement: Supplementary file 2 — Reporting Summary [file 41467_2025_66534_MOESM2_ESM.pdf]

nature portfolio

Corresponding author(s): Patricia Opresko

Last updated by author(s): Aug 25, 2025

Reporting Summary

Nature Portfolio wishes to improve the reproducibility of the work that we publish. This form provides structure for consistency and transparency in reporting. For further information on Nature Portfolio policies, see our Editorial Policies and the Editorial Policy Checklist.

Statistics

For all statistical analyses, confirm that the following items are present in the figure legend, table legend, main text, or Methods section.

n/a	Confirmed	
☐	☒	The exact sample size (*n*) for each experimental group/condition, given as a discrete number and unit of measurement
☐	☒	A statement on whether measurements were taken from distinct samples or whether the same sample was measured repeatedly
☐	☒	The statistical test(s) used AND whether they are one- or two-sided<br>*Only common tests should be described solely by name; describe more complex techniques in the Methods section.*
☒	☐	A description of all covariates tested
☒	☐	A description of any assumptions or corrections, such as tests of normality and adjustment for multiple comparisons
☐	☒	A full description of the statistical parameters including central tendency (e.g. means) or other basic estimates (e.g. regression coefficient) AND variation (e.g. standard deviation) or associated estimates of uncertainty (e.g. confidence intervals)
☐	☒	For null hypothesis testing, the test statistic (e.g. *F*, *t*, *r*) with confidence intervals, effect sizes, degrees of freedom and *P* value noted<br>*Give P values as exact values whenever suitable.*
☒	☐	For Bayesian analysis, information on the choice of priors and Markov chain Monte Carlo settings
☒	☐	For hierarchical and complex designs, identification of the appropriate level for tests and full reporting of outcomes
☒	☐	Estimates of effect sizes (e.g. Cohen's *d*, Pearson's *r*), indicating how they were calculated

Our web collection on statistics for biologists contains articles on many of the points above.

Software and code

Policy information about availability of computer code

Data collection	Nikon NIS Elements AR 5.02.01 for miscroscopy images, Labview software for smFRET experiments,
Data analysis	GraphPad Prism Software, version 10, MATLAB

For manuscripts utilizing custom algorithms or software that are central to the research but not yet described in published literature, software must be made available to editors and reviewers. We strongly encourage code deposition in a community repository (e.g. GitHub). See the Nature Portfolio guidelines for submitting code & software for further information.

Data

Policy information about availability of data

All manuscripts must include a data availability statement. This statement should provide the following information, where applicable:

- Accession codes, unique identifiers, or web links for publicly available datasets
- A description of any restrictions on data availability
- For clinical datasets or third party data, please ensure that the statement adheres to our policy

All data generated for this study are include in this article and its supplementary information files

Research involving human participants, their data, or biological material

Policy information about studies with human participants or human data. See also policy information about sex, gender (identity/presentation), and sexual orientation and race, ethnicity and racism.

Reporting on sex and gender	No sex- and gender-based analyses have been performed in this study since this is not applicable to the biochemistry and biophysical analyses performed. This is also not relevant to the cellular studies since 6-thio-dG inhibition of telomerase and telomere maintenance is not expected to be sex or gender specific.
Reporting on race, ethnicity, or other socially relevant groupings	N/A
Population characteristics	N/A
Recruitment	N/A
Ethics oversight	N/A

Note that full information on the approval of the study protocol must also be provided in the manuscript.

Field-specific reporting

Please select the one below that is the best fit for your research. If you are not sure, read the appropriate sections before making your selection.

☒ Life sciences ☐ Behavioural & social sciences ☐ Ecological, evolutionary & environmental sciences

For a reference copy of the document with all sections, see nature.com/documents/nr-reporting-summary-flat.pdf

Life sciences study design

All studies must disclose on these points even when the disclosure is negative.

Sample size	Sample size is shown in the figure or reported in the figure legend for each experiment
Data exclusions	No data were excluded from analysis
Replication	Each experiment was replicated at least 3 times, unless otherwise noted in the figure legend.
Randomization	Samples were allocated into the group depending on cell treatments as described in the manuscript. Randomization was not employed during these experiments because of the relatively small group numbers
Blinding	Where possible (e.g. immunofluorescence, FISH, metaphase analyses) were scored blindly. For other experiments (e.g. cell counts, colony formation, biochemistry) blinding was not possible because samples were loaded on slides or gels, or cells were seeded in dishes, in logical ways that require the experimentalist to know the treatment conditions.

Reporting for specific materials, systems and methods

We require information from authors about some types of materials, experimental systems and methods used in many studies. Here, indicate whether each material, system or method listed is relevant to your study. If you are not sure if a list item applies to your research, read the appropriate section before selecting a response.

Materials & experimental systems

n/a	Involved in the study	
☐	☒	Antibodies
☐	☒	Eukaryotic cell lines
☒	☐	Palaeontology and archaeology
☒	☐	Animals and other organisms
☒	☐	Clinical data
☒	☐	Dual use research of concern
☒	☐	Plants

Methods

n/a	Involved in the study	
☒	☐	ChIP-seq
☒	☐	Flow cytometry
☒	☐	MRI-based neuroimaging

Antibodies

Antibodies used	Anti-gammaH2AX (Ser139) mouse monoclonal Santa Cruz Cat#sc-517348 p-histone-h2a-x-antibody ser-139; RRID:AB_2783871
Validation	The anitbodies were validated by the manufacturer. Santa Cruz: "Santa Cruz Biotechnology is committed to providing the highest

level of quality and service". https://www.scbt.com/customer-care/terms

Eukaryotic cell lines

Policy information about cell lines and Sex and Gender in Research

Cell line source(s)	RPE1-hTERT, U2OS, HCT-116 and HEK293-T were from ATCC. HeLa LT and HeLa VST were a generous gift from Dr. Roderick O'Sullivan (University of Pittsburgh), and HEK293-FT were from ThermoFisher. Cell treatments with 6-thio-dG were as indicated in the figure legends
Authentication	Cell lines were authenticated by ATCC.
Mycoplasma contamination	Cell lines are tested for mycoplasma contamination using the ATCC Universal Mycoplasma Detection Kit to confirm that they test negative for mycoplasma infection. The HCT116 cells were tested by DAPI staining and microscopy of fixed cells
Commonly misidentified lines (See ICLAC register)	No commonly misidentified lines were used in this study.

Plants

Seed stocks	*Report on the source of all seed stocks or other plant material used. If applicable, state the seed stock centre and catalogue number. If plant specimens were collected from the field, describe the collection location, date and sampling procedures.*
Novel plant genotypes	*Describe the methods by which all novel plant genotypes were produced. This includes those generated by transgenic approaches, gene editing, chemical/radiation-based mutagenesis and hybridization. For transgenic lines, describe the transformation method, the number of independent lines analyzed and the generation upon which experiments were performed. For gene-edited lines, describe the editor used, the endogenous sequence targeted for editing, the targeting guide RNA sequence (if applicable) and how the editor was applied.*
Authentication	*Describe any authentication procedures for each seed stock used or novel genotype generated. Describe any experiments used to assess the effect of a mutation and, where applicable, how potential secondary effects (e.g. second site T-DNA insertions, mosiacism, off-target gene editing) were examined.*
